# Advancements in Predicting Periodontal Disease Evolution: A Narrative Review of Contemporary Clinical Risk Prediction Systems

**DOI:** 10.7759/cureus.99164

**Published:** 2025-12-13

**Authors:** Shankar S Menon, Arun Kurumathur Vasudevan

**Affiliations:** 1 Periodontics, Amrita School of Dentistry, Amrita Vishwa Vidyapeetham, Kochi, IND

**Keywords:** periodontal risk factors, precision periodontics, risk assessment, risk assessment tools, risk prediction

## Abstract

Periodontal diseases are multifactorial chronic inflammatory disorders characterised by progressive destruction of the tooth-supporting apparatus. Predicting the evolution of these diseases remains a significant clinical challenge because microbial, host, behavioral, and systemic determinants interact to produce substantial inter-individual variability. In recent decades, periodontal care has transitioned from a reactive model toward a preventive, risk-oriented approach supported by structured prediction systems. Various clinical risk assessment tools, including the hexagonal periodontal risk assessment (PRA), modified PRA (MPRA), UniFe/PerioRisk, SmartRisk, DentoRisk, and the Periodontal Risk Calculator (PRC/PreViser), have been designed to quantify and visualise an individual’s susceptibility to disease progression. These models integrate multiple parameters such as probing depth, bleeding on probing, bone loss, smoking, diabetes, and host-response markers to generate patient- or tooth-level prognoses. Recent developments have incorporated artificial intelligence-driven algorithms and real-time digital data capture to enhance predictive accuracy and facilitate personalised maintenance strategies. This narrative review critically analyses the evolution, structure, validation evidence, and clinical applicability of major periodontal risk prediction systems and explores future directions for integrating biological, behavioral, and digital determinants to achieve precision periodontics.

## Introduction and background

Periodontal diseases remain among the most prevalent chronic inflammatory conditions worldwide and are a principal cause of tooth loss in adults. Although bacterial plaque is the initiating aetiologic factor, the progression of disease is influenced by host susceptibility, behavioural exposures, and systemic health, resulting in heterogeneous clinical manifestations among individuals with similar microbial burdens. Conventional diagnostic indices such as probing depth, clinical attachment loss, and radiographic bone loss describe historical tissue destruction but fail to predict future disease progression. To address this limitation, structured risk assessment has been proposed as a complement to diagnosis and treatment planning [1].

Risk assessment in periodontology involves identifying major modifiable risk factors including tobacco smoking (which causes dose-dependent impairment of healing and immunity), poor oral hygiene, uncontrolled diabetes mellitus (via neutrophil dysfunction), psychological stress, and infrequent dental visits; and non-modifiable or partially modifiable factors which comprise age, genetic polymorphisms (e.g., interluekin-1), male sex, osteoporosis, obesity/metabolic syndrome, rheumatoid arthritis, cardiovascular disease, chronic kidney disease, and certain medications (e.g., phenytoin, calcium-channel blockers), all of which amplify inflammatory response or reduce tissue repair capacity. Evaluating the cumulative impact of these variables enables clinicians to categorise patients into risk levels and individualise therapy and recall intervals [2].

The pathogenesis of periodontitis in the context of behavioural exposures and systemic comorbidities is best conceptualised as a two-step process of microbial dysbiosis followed by an aberrant host response. Habitual exposures such as cigarette smoking alter the subgingival microbiome towards a more proteolytic, anaerobic consortium while simultaneously impairing neutrophil chemotaxis, reducing gingival blood flow and modifying local immune surveillance; these effects together facilitate deeper pocket formation and increased connective tissue breakdown. Poor glycemic control in diabetes fosters the formation of advanced glycation end products that modify extracellular matrix proteins and enhance proinflammatory signalling, which accelerate collagen degradation and bone resorption. Psychological stress activates the hypothalamic-pituitary-adrenal axis and sympathetic pathways, increasing circulating glucocorticoids and catecholamines, which suppress certain arms of the immune system and can promote dysbiotic shifts in the oral microbiome. Medications (for example, phenytoin, cyclosporine, calcium channel blockers) may exacerbate gingival overgrowth and impede plaque control, thereby increasing local inflammatory stimulus. Highlighting these mechanistic links provides the biological rationale for why specific systemic illnesses and habits are included as predictors in risk models and supports targeted therapeutic modification [3].

The American Academy of Periodontology (AAP) and the European Federation of Periodontology (EFP) have emphasised incorporation of formal risk assessment as a cornerstone of contemporary periodontal management [4]. The conceptual foundation of clinical risk prediction was established with the introduction of the periodontal risk assessment (PRA) model by Lang and Tonetti in 2003, which employed a hexagonal vector diagram to represent six principal parameters influencing disease progression [5]. Chandra’s modified PRA (MPRA) in 2007 expanded upon this model by introducing numerical scoring and including additional variables such as tooth loss and inflammatory burden [6]. Subsequently, Trombelli and co-workers developed the UniFe/PerioRisk model in 2009, designed for epidemiologic and clinical application, followed by SmartRisk in 2017, an artificial-intelligence (AI)-driven platform capable of dynamic recalibration through longitudinal data input [7,8]. DentoRisk, proposed by Lindskog et al. in 2010, refined the approach by assessing both patient- and tooth-level risk through algorithmic computation [9]. The Periodontal Risk Calculator (PRC/PreViser), introduced by Page et al., similarly utilised a digital algorithm integrating clinical and systemic parameters to generate quantitative risk scores [10]. These systems collectively marked a paradigm shift from descriptive diagnosis toward predictive, preventive, and personalised periodontics.

Despite these advancements, inconsistencies persist regarding variable selection, weighting algorithms, and external validation across diverse populations [11]. Therefore, a comprehensive appraisal of their methodological foundations, accuracy, and clinical practicality is warranted. The following sections provide an evidence-based narrative synthesis of the principal periodontal risk-prediction systems, including detailed discussion and tabulated comparison of their parameters, scoring logic, and validation outcomes.

## Review

Hexagonal risk diagram for PRA

The PRA model introduced by Lang and Tonetti in 2003 represented the first structured attempt to quantify and visualise patient-level risk for periodontitis progression during supportive periodontal therapy. The system incorporated six major parameters: percentage of bleeding on probing, number of sites with probing depth ≥ 5 mm, bone loss relative to age, number of lost teeth, smoking status, and presence of systemic or genetic factors such as diabetes or interleukin-1 gene polymorphism [12]. Each parameter was plotted on one of six axes radiating from the centre of a hexagon, generating a “spider-web” diagram whose size and area corresponded to overall risk magnitude. Patients whose plots occupied the innermost region were considered low-risk, whereas those extending toward the outer sectors were categorised as moderate- or high-risk depending on the number of parameters lying in the outer zones [5].

The conceptual strength of the PRA model lies in its holistic and easily interpretable graphical display, which allows simultaneous visualisation of multiple determinants. By identifying modifiable parameters such as smoking or residual pockets, clinicians can motivate patient engagement and customise recall intervals, typically 6-12 months for low-risk, 3-6 months for moderate-risk, and 1-3 months for high-risk individuals. Subsequent longitudinal studies by Lang et al. and Tonetti et al. demonstrated significant associations between the PRA-defined high-risk category and increased tooth loss during maintenance therapy [9,13]. Reproducibility analyses indicated good inter-examiner agreement when parameters were consistently defined, although the subjective estimation of bone-loss-to-age ratio occasionally introduced variability. The principal limitation of the PRA is its semi-quantitative nature: categorical grading of each factor restricts numerical precision and limits statistical modelling of risk probabilities. Furthermore, the model does not accommodate temporal changes in risk parameters, rendering it essentially static.

Despite these shortcomings, the PRA remains one of the most widely applied chair-side frameworks for risk communication and longitudinal monitoring, particularly in Europe, where it has been endorsed by the European Federation of Periodontology [4]. Its enduring influence is reflected in later models that either adapted its variables or sought to replace its qualitative scoring with numerical computation. The parameters of the model are described in Table 1.

**Table 1:** Lang and Tonetti’s Periodontal Risk Assessment (PRA) Model SPT: Supportive Periodontal Therapy, IL-1: Interleukin-1 Table credits: Dr. Shankar S Menon

Parameter	Measurement/Scoring	Risk Categories	Validation Evidence
Bleeding on probing (%)	0–9 = low; 10–25 = moderate; > 25 = high	Incorporated into hexagonal vector	Correlated with recurrence of inflammation during SPT
Sites with probing depth ≥ 5 mm	Count of pockets per dentition	≥ 4 sites = high risk	Strong predictor of tooth loss
Bone loss/age ratio	Mean radiographic bone loss (%) ÷ age (years)	> 1 = high risk	Associated with progression rate
Number of lost teeth	Excluding third molars	≥ 8 = high risk	Reflects past disease experience
Smoking status	Non-, former-, or current smoker	Weighted qualitatively	Dose-response relationship confirmed
Systemic factors	Diabetes, IL-1 polymorphism etc.	Present = high risk	Predictive in cohort studies

Modified periodontal risk assessment model

Recognising the need for increased objectivity, Chandra in 2007 proposed the MPRA model, which retained the multifactorial structure of Lang and Tonetti’s PRA but introduced a numerical scoring algorithm. This model incorporated both clinical and systemic factors, bleeding on probing, number of sites with probing depth ≥ 5 mm, clinical attachment loss-to-age ratio, tooth loss, smoking, diabetes mellitus, and stress levels, and assigned each a weighted score from 0 to 5 based on severity thresholds. The cumulative mean score was then used to classify patients into low (< 2), moderate (2-4), or high (> 4) risk categories [6].

The inclusion of quantitative weights enabled statistical analysis of continuous data and improved inter-observer reproducibility. Furthermore, by incorporating inflammatory burden and tooth loss, the MPRA linked both historical and current disease activity to systemic conditions, thus bridging the gap between clinical parameters and overall health status. A prospective validation of Indian patients showed a significant correlation between MPRA scores and clinical attachment loss after 12 months of maintenance [14]. However, this model also presents limitations. Its higher number of variables makes it time-consuming in routine settings, and the weighting system has not been externally standardised. Regional derivation without large multicentre validation restricts its generalisability beyond the population in which it was developed. Nonetheless, the MPRA remains a valuable educational and clinical tool, particularly in academic institutions where comprehensive risk profiling is feasible. The parameters of the model are described in Table 2.

**Table 2:** Chandra’s Modified Periodontal Risk Assessment (MPRA) CAL: Clinical Attachment Level Table credits: Dr. Shankar S Menon

Parameter	Scoring (0–5)	Risk Contribution	Key Evidence
Bleeding on probing (%)	0 = < 10%; 5 = > 50%	Reflects inflammatory burden	Prospective link with disease recurrence
Sites with probing depth ≥ 5 mm	0 = none; 5 = > 10 sites	Indicates residual pockets	Predictor of attachment loss
CAL/age ratio	0 = < 0.5; 5 = > 2	Captures cumulative destruction rate	Validated in a 12-month cohort
Tooth loss	0 = 0–2; 5 = > 8	Historical disease burden	Associated with future loss
Smoking (pack-years)	0 = nonsmoker; 5 = > 10 pack-years	Major modifiable risk factor	Dose-dependent effect
Diabetes status (HbA1c %)	0 = ≤ 6; 5 = ≥ 8	Systemic modifier of inflammation	Well-established link
Socioeconomic factors	0–5 graded scale	Behavioural component of risk	Correlated with treatment outcome

UniFe/PerioRisk model

In 2009, Trombelli and colleagues at the University of Ferrara introduced the UniFe or PerioRisk model, which was subsequently refined for epidemiological and supportive-care contexts. This model aimed to simplify risk estimation while retaining prognostic validity by using only five major parameters: age, number of sites with probing depth ≥ 5 mm, bleeding on probing, bone-loss-to-age ratio, and systemic factors (smoking and diabetes). Each parameter was allocated a discrete score (0-2), and the aggregate value placed the patient into one of five categories ranging from 1 (lowest risk) to 5 (highest risk). UniFe/PerioRisk was designed to be applied at the conclusion of active periodontal therapy to predict the likelihood of future disease progression during supportive periodontal care [7].

Long-term validation studies involving over 200 patients demonstrated that higher PerioRisk classes were significantly associated with increased tooth-loss rates during 10 years of maintenance [15]. The model’s simplicity facilitated integration into routine practice, and its categorical scheme allowed easy communication with patients. However, PerioRisk has been criticised for its relatively coarse scoring intervals and reduced sensitivity to subtle changes within individual patients. Furthermore, validation cohorts were limited to European populations, warranting further research across diverse ethnic and socio-economic groups. Nevertheless, comparative analyses have shown PerioRisk to perform as well as or better than more complex algorithms such as the Periodontal Risk Calculator in predicting tooth loss during supportive care. Recent adaptations incorporated the model into digital platforms such as SmartRisk, extending its applicability in technology-enhanced environments [8]. The parameters of the model are described in Table 3.

**Table 3:** UniFe/PerioRisk Model Table credits: Dr. Shankar S Menon

Parameter	Score Range	Risk Category (1–5)	Validation Highlights
Age (years)	0 = < 40; 1 = 40–60; 2 = > 60	Weight for cumulative exposure	Correlated with annual tooth loss
Probing depth ≥ 5 mm (sites)	0 = 0–3; 1 = 4–8; 2 = > 8	Indicator of residual disease	Significant predictor
Bleeding on probing (%)	0 = < 10; 1 = 10–25; 2 = > 25	Reflects current inflammation	Associated with recurrence
Bone loss/age ratio	0 = < 0.5; 1 = 0.5–1; 2 = > 1	Measures disease severity	Linked to future progression
Systemic factors	0 = absent; 2 = present	Modifiers of risk response	Enhanced predictive accuracy

Collectively, these three classical models, PRA, MPRA, and UniFe/PerioRisk, represent progressive milestones in the conceptual evolution of periodontal risk assessment [16]. The PRA introduced multifactorial visualisation; the MPRA added numerical precision and systemic integration; and UniFe/PerioRisk provided operational simplicity and longitudinal validation. Each has contributed to embedding risk-based reasoning into periodontal practice and to framing subsequent algorithmic and digital innovations that now define precision periodontology.

SmartRisk model

Building upon the PerioRisk framework, Trombelli et al. introduced the SmartRisk model in 2017 as a digital evolution of UniFe/PerioRisk designed to exploit electronic data and AI computation for continuous recalibration. The model integrates classical variables-probing depth ≥ 5 mm, bleeding on probing, bone-loss-to-age ratio, smoking, and diabetes-but operates within an adaptive statistical environment that updates risk weights as new patient data are entered. SmartRisk employs a Bayesian-type learning algorithm that allows real-time adjustment of prior probabilities, thereby accommodating changes in periodontal and systemic status during supportive care [8].

Clinically, SmartRisk displays a dashboard-based output with colour-coded risk zones corresponding to the patient’s longitudinal data. This provides clinicians with predictive trajectories rather than static snapshots. Validation studies demonstrated good discriminatory ability for tooth-loss outcomes compared with static PerioRisk categories, particularly when longitudinal inflammatory parameters were included [16,17].

SmartRisk is characterised by several distinguishing features that set it apart from static, point-in-time risk instruments. First, SmartRisk operates as an adaptive, data-driven platform intended to integrate longitudinal clinical inputs rather than a single cross-sectional snapshot; probing depths, bleeding indices, radiographic bone measurements, and systemic parameters are updated across maintenance visits, and the model recalibrates risk weights accordingly. Second, SmartRisk employs probabilistic or Bayesian updating to refine prior risk estimates as new evidence accumulates, yielding predictive trajectories that reflect both baseline susceptibility and recent treatment response. Third, the platform is engineered for interoperability with electronic health records, allowing automated extraction of demographic and medical data and reducing manual entry burden. Fourth, its user interface typically presents risk as a dynamic dashboard with colour-coded zones and trend plots, facilitating clinician interpretation and patient communication. Fifth, SmartRisk can incorporate variable granularity ranging from patient-level risk to tooth-level alerts depending on the deployed algorithm. Finally, SmartRisk emphasises explainability by providing clinicians with the relative contribution of key predictors to an individual’s risk score, thereby supporting clinical reasoning and informing tailored interventions. These features together enhance the model’s utility for monitoring, prognostication, and decision support in routine practice, particularly where repeated measures are available. SmartRisk thus represents a conceptual shift from risk assessment to risk prediction, embedding temporal data analysis and personalised feedback mechanisms into periodontal maintenance [8]. The parameters of the model are described in Table 4.

**Table 4:** SmartRisk Model Table credits: Dr. Shankar S Menon

Parameter	Input Type	Dynamic Adjustment	Validation Highlights
Probing depth ≥ 5 mm	Continuous	Weight recalibrated after each visit	Predictive of tooth loss
Bleeding on probing (%)	Continuous	Weighted against baseline	Significant in longitudinal regression
Bone loss/age ratio	Continuous	Auto-updated by radiographic data	Correlated with progression
Smoking status	Binary/dose	Bayesian update on cessation	Reduced risk after quitting
Diabetes (HbA1c)	Continuous	Adjusted by metabolic control	Independent modifier

DentoRisk system

Lindskog et al. (2010) developed the DentoRisk system, a computer-based algorithm capable of estimating both patient- and tooth-level risk by integrating epidemiologic and longitudinal data. The model employs logistic-regression equations derived from prospective cohorts in which variables such as age, smoking, bleeding on probing, tooth mobility, pocket depth, bone loss, and systemic health are entered into probability functions generating individual risk values (0-1). The system can thus assign specific probabilities of tooth loss or disease progression per tooth, making it unique among clinical tools [18].

Validation across Swedish maintenance populations confirmed that DentoRisk achieved an area-under-the-curve (AUC) of 0.83 for predicting tooth loss, surpassing classical staging-grading metrics [19]. Subsequent multicentre evaluations reaffirmed the algorithm’s performance, especially when variables were updated annually [20]. The model’s main limitation lies in its dependence on extensive data entry and the requirement for calibration of radiographic and probing assessments, which may restrict use in general practice. Nonetheless, it remains a robust prognostic instrument for both clinical and research purposes, enabling prioritisation of teeth and patients according to quantified risk values. The parameters of the model are described in Table 5.

**Table 5:** DentoRisk System AUC: Area-under-the-curve Table credits: Dr. Shankar S Menon

Level	Predictor Variables	Output Metric	Validation Outcomes
Patient level	Age, smoking, diabetes, mean bone loss, residual pockets	Probability (0–1) of tooth loss per year	AUC 0.83 in 10-year follow-up
Tooth level	Mobility, bone loss %, pocket depth, position	Probability (0–1) of tooth loss	High accuracy for molars
Update interval	Annually	Dynamic recalculation	Improved prediction with updated data

Periodontal risk calculator (PRC/PreViser)

Page et al. introduced the Periodontal Risk Calculator (PRC) in 2002, later commercialised as the PreViser Risk Calculator, representing one of the earliest web-based algorithmic risk-assessment systems. The PRC computes a numeric risk score (0-100) based on eleven clinical and systemic variables, including age, smoking, diabetes, systemic health, pocket depth, bleeding on probing, furcation involvement, tooth loss, and bone loss. It was designed for chair-side use and automatic generation of risk reports for patient education [10].

Clinical validation demonstrated a significant correlation between higher PRC scores and subsequent attachment loss over five years [21]. Comparative analyses with the PRC and the PRA, explained earlier, showed PRC to be an algorithmic, numerical calculator that synthesises multiple quantitative variables-including age, smoking exposure, systemic health, pocket depths, bleeding, furcation involvement and radiographic bone loss-to generate a continuous risk score and a corresponding severity index; while in contrast, PRA has been seen to be a graphical, semi-quantitative instrument that maps six core parameters onto a hexagonal spider diagram and classifies patients into low, moderate or high risk categories: its strength lies in intuitive visual communication and ease of interpretation during a chair-side examination. In terms of performance, PRC generally offers greater granularity and objective numeric comparability across patients, whereas PRA provides a rapid, visually intuitive assessment that is well suited for patient counselling. PRC’s disadvantages include dependence on proprietary algorithms and potentially greater data entry time, while PRA’s limitations include coarser categorical thresholds and limited capacity for fine-grained statistical modeling. Both instruments can be complementary: PRA is useful for immediate clinical triage and patient education, whereas PRC serves well when detailed quantification, longitudinal monitoring, or integration into quality-assurance systems is required. The choice between them should therefore be guided by clinic workflow, the need for numeric precision, and the availability of digital infrastructure [15]. Another limitation with PRC is the dependence on proprietary software and region-specific calibration data, which may limit global generalizability. Nevertheless, the PRC remains a landmark development linking clinical input directly to quantified risk outputs and has inspired later AI-enabled systems.

AAP risk-assessment tool

The American Academy of Periodontology (AAP) Risk-Assessment Tool was developed as a simplified clinical instrument for routine use, emphasising modifiable risk factors accessible without computational software. It evaluates age, smoking, diabetes, oral hygiene, and history of periodontal disease, each rated as low, moderate, or high. A cumulative profile yields an overall qualitative risk category [22]. Although not algorithmic, this approach aligns with public-health objectives by facilitating rapid patient education and engagement.

Studies assessing its reproducibility revealed fair-to-moderate inter-examiner agreement (κ = 0.61) and a strong predictive association between high AAP-risk category and tooth loss during maintenance [23]. However, due to its simplicity, it lacks the granularity required for research or advanced clinical decision-support systems. Its major advantage lies in accessibility, serving as an entry-level screening framework for general practitioners. The parameters of the model are described in Table 6.

**Table 6:** AAP Risk-Assessment Tool Table credits: Dr. Shankar S Menon

Factor	Grading	Risk Interpretation	Supporting Evidence
Smoking	None/ < 10/ > 10 pack-years	Strong modifier	Correlated with tooth loss
Diabetes	Controlled/uncontrolled	Systemic risk	Predicts bone loss
Plaque control	Good/fair/poor	Behavioural determinant	Associated with recurrence
Past periodontal history	None/moderate/severe	Cumulative exposure	Validated in maintenance cohorts

HIDEP and BEDS CHASM frameworks

The HIDEP (Health Improvement through Dental Evaluation and Prevention) and BEDS CHASM (Bleeding, Extent, Depth, Systemic conditions, Calculus, History, Age, Smoking, Maintenance) models were designed primarily for epidemiologic risk surveillance rather than individualised prediction [24,25]. Both frameworks emerged from large community-based datasets aiming to stratify periodontal risk at the population level using simple categorical indices. HIDEP aggregates periodontal parameters into a composite score (0-5) weighted by systemic modifiers [24], while BEDS CHASM employs a matrix-based scoring integrating eight easily recorded variables [25].

Although less detailed than clinic-based tools, these models have been valuable for health-policy applications by identifying high-risk clusters within populations and enabling resource allocation. Validation has shown moderate correlation between BEDS CHASM scores and clinical attachment loss, supporting its use in epidemiologic monitoring [25].

RABIT (risk algorithm-based individualised therapy) system

The RABIT system, developed by Teich ST, exemplifies next-generation computational risk prediction integrating clinical, microbiologic, and host-response biomarkers. Using multivariate logistic regression and machine-learning classifiers, RABIT predicts probabilities of progression events such as new attachment loss ≥ 2 mm within 12 months. Predictor sets include pocket depth distribution, bleeding on probing, subgingival microbial composition, cytokine profile, smoking, and glycaemic status [26].

RABIT exemplifies an advanced prognostic framework that integrates clinical, microbiologic and host-response biomarkers using multivariate and machine-learning classifiers. Its principal advantages include enhanced predictive accuracy because it combines orthogonal data streams and the ability to produce individualized probabilities of progression that can guide highly tailored therapeutic decisions. RABIT’s incorporation of molecular markers and pathogen quantification can identify subgroups who may benefit from adjunctive antimicrobials, host-modulation therapy, or more aggressive surgical intervention. Additionally, RABIT’s dynamic updating facilitates monitoring of treatment response and risk reduction over time. However, RABIT has notable disadvantages. The requirement for laboratory assays and molecular diagnostics increases cost and complexity and limits immediate applicability in low-resource settings. There are also implementation challenges related to data standardisation, assay variability, the need for large training datasets to avoid overfitting, and potential algorithmic bias if the training cohorts are not sufficiently diverse. Data privacy, explainability of complex machine-learning models, and the regulatory framework for clinical decision support systems are further concerns. Thus, robust external validation across multiple populations is necessary before RABIT can be recommended for routine care outside research environments [26].

Pilot validation on 300 patients demonstrated an AUC of 0.87 for progression prediction, outperforming PRA and PerioRisk in the same cohort [15,16]. Importantly, RABIT can auto-update through iterative model training, a hallmark of AI-enabled precision periodontics. Its limitation lies in the requirement for biomarker assays not yet routinely available in all practices. Nonetheless, RABIT illustrates how integration of molecular data can substantially enhance predictive precision. The parameters of the model are described in Table 7.

**Table 7:** RABIT System AUC: Area-under-the-curve, P. gingivalis: Porphyromonas gingivalis, T. forsythia: Tannerella forsythia, IL-1β: Interleukin-1 beta, TNF-α: Tumor Necrosis Factor-alpha, MMP-8: Matrix Metalloproteinase-8 Table credits: Dr. Shankar S Menon

Predictor Categories	Examples	Statistical Engine	Validation Metrics
Clinical	Pocket depth, bleeding, mobility	Machine learning	AUC 0.87 for progression
Microbial	P. gingivalis, T. forsythia load	Random-forest weighting	Enhanced discrimination
Host biomarkers	IL-1β, TNF-α, MMP-8 levels	Bayesian integration	Improved precision
Systemic factors	Smoking, diabetes, compliance	Dynamic updating	Real-time recalibration

Synthesis with modern approaches involving AI

Clinical risk-prediction models assist clinicians through structured stratification of patient and tooth risk, enabling personalised decisions on intensity and timing of interventions. At the initial assessment, risk models identify patients who require intensive active therapy and close follow-up versus those who can be managed with conservative therapy and standard recall intervals. For instance, a high risk score may prompt escalation from nonsurgical scaling and root planning to adjunctive systemic or local antimicrobials, incorporation of host-modulatory agents, earlier consideration of surgical access or regenerative procedures for deep defects, or tooth-level prioritisation for extraction versus restorative attempts. During maintenance, models inform recall intervals: low-risk patients may safely extend recall to longer intervals, while high-risk patients benefit from shorter recall and more frequent reinforcement of oral hygiene. At the tooth level, model outputs can prioritise molars or teeth with high site-level probability of progression for targeted therapy, thereby optimising resource allocation. Importantly, models can improve patient communication by converting abstract risk into actionable targets (for example, smoking cessation or achieving glycemic control) and by documenting objective rationale for treatment intensity, which has medicolegal and quality-assurance benefits. To translate predictive insight into improved outcomes, clinicians must integrate model outputs with clinical judgment, consider patient preferences and comorbidities, and ensure consistent reassessment and calibration of the chosen model in their practice setting.

In a 2023 study, Patel et al. developed a computerized clinical decision-support system (CDSS) named Perio-Risk Scoring System (PRSS) intended to help clinicians generate periodontal risk scores, provide diagnoses, and identify which factors most influence periodontal risk and prognosis. In their report, they described how PRSS offers a structured, reproducible way to integrate multiple patient- and disease-related parameters into a unified “perio-score,” supporting clinical decision-making. The study suggested that such a CDSS can improve consistency in periodontal assessments and help highlight key risk contributors across patients [27].

A 2025 study suggested a new algorithm/tool for periodontal risk assessment, diagnosis and prognosis. This was named GF-PeDRA©: Global Factors - Periodontal Diagnosis, Risk Assessment, and Assessment of Prognosis. This was a clinical study with 221 patients, combining 18 clinical, radiographic and patient-related factors (like probing depth, bone loss, bleeding on probing, smoking, diabetes, age, among others) to produce a score - expressed as a percentage of an “octadecagon area” - which classifies prognosis from “good” to “hopeless.” The automated diagnoses produced by GF-PeDRA matched professional clinical diagnoses perfectly, suggesting that the algorithm is reliable. Based on the scores, patients were distributed across prognostic categories: ~21.7% good, ~19.5% fair, ~19.5% poor, ~30.8% questionable and ~8.6% hopeless prognosis [28].

Collectively, these modern and AI-driven systems signify a major paradigm shift in periodontal prognostics. Whereas classical models such as PRA and MPRA relied on static categorical inputs, contemporary frameworks like SmartRisk, DentoRisk, and RABIT (as well as new studies devising newer tools like GF-PeDRA© which require further clinical validation), incorporate longitudinal data streams and computational learning to produce dynamic, patient-specific predictions [15,16]. Validation evidence supports incremental improvements in accuracy, though external replication remains essential. Importantly, the growing ability to integrate microbiologic and host-response data heralds a transition toward precision periodontics, wherein preventive schedules and therapeutic intensity are tailored to each patient’s biologic and behavioural profile.

Despite technological progress, several challenges persist: heterogeneity in outcome definitions, limited cross-population calibration, and concerns about algorithm transparency and data privacy. Future models must balance complexity with clinical usability, ensuring that predictive insights translate into tangible improvements in patient outcomes.

## Conclusions

The evolution of periodontal risk-prediction systems reflects a fundamental transformation in periodontal care from a reactive, descriptive model of diagnosis to a proactive, precision-based framework centred on prevention. The classical PRA established the conceptual foundation for visualising risk through multifactorial parameters, while subsequent models such as the MPRA and UniFe/PerioRisk enhanced numerical precision and simplified applicability. Modern algorithmic and digital systems, including SmartRisk, DentoRisk, and the Periodontal Risk Calculator, further advanced this trajectory by incorporating longitudinal data, automated computation, and dynamic calibration.

Emerging AI-driven frameworks such as RABIT exemplify the integration of clinical, microbial, and host-response biomarkers, heralding an era of predictive periodontics aligned with personalised medicine. Despite notable progress, challenges remain regarding external validation, algorithm transparency, and equitable clinical implementation across populations. Future research must emphasise large-scale, multicentre validation and the ethical application of AI to ensure that predictive models translate into measurable improvements in periodontal stability, tooth retention, and overall patient well-being. Ultimately, advancements in risk-prediction systems mark a decisive step toward individualised, evidence-based periodontal care that aligns preventive strategies with each patient’s unique biological and behavioural profile.
